# Are the special educational needs of children in their first year in primary school in Ireland being identified: a cross-sectional study

**DOI:** 10.1186/1471-2431-14-52

**Published:** 2014-02-19

**Authors:** Margaret Curtin, Denise Baker, Anthony Staines, Ivan J Perry

**Affiliations:** 1 Department of Epidemiology and Public Health, University College Cork, Floor 4, Western Gateway Building, Cork, Ireland; 2 School of Nursing and Human Sciences, Dublin City University, Dublin 9, Ireland

**Keywords:** Child development, Special educational needs, Population-health, Social determinants of health, Educational needs assessment

## Abstract

**Background:**

If the window of opportunity presented by the early years is missed, it becomes increasingly difficult to create a successful life-course. A biopsychosocial model of special educational need with an emphasis on participation and functioning moves the frame of reference from the clinic to the school and the focus from specific conditions to creating supportive environments cognisant of the needs of all children. However, evidence suggests that an emphasis on diagnosed conditions persists and that the needs of children who do not meet these criteria are not identified.

The Early Development Instrument (EDI) is a well-validated, teacher-completed population-level measure of five domains of child development. It is uniquely placed, at the interface between health and education, to explore the developmental status of children with additional challenges within a typically developing population. The aim of this study was to examine the extent to which the special educational needs of children in their first year of formal education have been identified.

**Methods:**

This cross-sectional study was conducted in Ireland in 2011. EDI (teacher completed) scores were calculated for 1344 children. Data were also collected on special needs and on children identified by the teacher as needing assessment. Mean developmental scores were compared using one-way ANOVA.

**Results:**

Eighty-three children in the sample population (6.2%) had identified special educational needs. A further 132 children were judged by the teacher as needing assessment. Children with special needs had lower mean scores than typically developing children, in all five developmental domains. Children considered by the teacher as needing assessment also had lower scores, which were not significantly different from those of children with special needs. Speech, emotional or behavioural difficulties were the most commonly reported problems among children needing further assessment. There was also a social gradient among this group.

**Conclusions:**

A small but significant number of children have not had their needs adequately assessed. Teacher observation is an effective means of identifying children with a level of impairment which prevents them from fully participating in their educational environment and could be integrated into a multi-disciplinary approach to meeting the needs of all children.

## Background

If the window of opportunity presented by the early years is missed, it becomes increasingly difficult, in terms of both time and resources, to create a successful life course [1]. The foundations for virtually every aspect of human development – physical, intellectual and emotional - are laid in early childhood [2]. Yet for many children developmental delay remains undetected until the formal education years leading to a greater risk of academic failure, behavioural problems and long term socio-economic disadvantage [3].

An understanding of child development as a social process of interaction between children and their environment [4] is compatible with a shift from a ‘medical’ to a ‘social’ understanding of disability and special educational needs [5]. A biopsychosocial model of child development with an emphasis on participation, functioning and the child’s ability to interact with their environment underpins the World Health Organisation’s International Classification of Functioning (ICF) Disability and Health [6] and has led to a shift from a deficit model of individual disability to a focus on inclusive education and interdisciplinary working between education, health and social services [7]. This moves the frame of reference from the clinic to the school and the focus from children identified through a standard, predominantly biomedical, framework to those identified by teachers as requiring additional support [8].

Children with special educational needs should be identified as early as possible. Early intervention is vital but to obtain this an early assessment is needed. Ideally children should be assessed in pre-school, as the earlier the assessment, the greater the chance he or she has of developing coping strategies [9].

In Ireland, the Education of Persons with Special Educational Needs (EPSEN) Act of 2004 provides a legislative underpinning for inclusive education for all children with an identified educational need, not confined to those with an identifiable disability or diagnosis [10]. However, the Irish systems and services have not changed in line with the act resulting in an emphasis on identified medical conditions instead of participation and functioning [11]. Children with less clearly defined needs are therefore less likely to benefit [12].

Distinction is also necessary between assessment for the purpose of identifying children’s learning needs and assessment for the purpose of resource allocation. Where this distinction becomes blurred, children are at risk of being prematurely labelled in an attempt to ensure that they qualify for support [13]. Qualitative studies suggest that, in Ireland, this emphasis on diagnosis persists [12,14,15].

### The Early Development Instrument

This study used the Early Development Instrument (EDI) to assess the development status of children in their first year of formal education [16]. The EDI is a well-validated, teacher-completed population level measure of five domains of child development at school entry age designed at the Offord Centre for Child Studies, McMasters University, Hamilton, Ontario in the late 1990s [17]. It is uniquely placed, at the interface between health and education, to explore the developmental status children with additional challenges in the context of a typically developing population. At the same time, the EDI is a population level measure and not a diagnostic tool. It is based on the premise that universal approaches work best in improving long term developmental outcome for all children and provides evidence to establish the incidence and distribution of developmental delay and to identify populations of children at greater risk [18].

The instrument consists of five domains and 104 questions. The domains are *Physical health and well-being* (fine an gross motor skills, physical readiness for the school day and child health); *Social competence* (self-confidence, ability to play, get along with others and share); *Emotional maturity* (ability to concentrate, help others, patient, not aggressive or angry); *Language and cognitive development* (interest in reading and writing, ability to count and recognise numbers and shapes); and *Communication skills and general knowledge* (ability to tell a story, communicate with adults and children, articulate themselves) [16].

This study, for the first time, within a typically developing Irish population, quantified the extent to which the special educational needs of children in their first year of formal education are being met. The aim of this study was to examine, at a population level, using EDI data, the extent to which children in their first year of formal education have their developmental and special educational needs identified.

## Methods

This cross-sectional study of child development using the EDI was implemented with children in their first year of formal education (in Ireland this is referred to as ‘Junior Infants’) in 42 out of 47 primary schools in Cork city in April/May 2011 and a further five schools in an adjoining rural community. Five schools in the city declined to participate. These declining schools were representative of a cross section of schools in the city and would not affect the composition of the study [16]. Parents of all eligible children in the participating schools were informed about the study and invited to have their child included. Eligibility criteria were: being in the latter half of the first year of formal education, being in the class more than one month and not having left the school.

Ireland is a largely homogenous country and Cork city is typical of the Irish urban population. Moreover, the education system is consistent throughout the country, with all schools adhering to nationally defined curriculum and standards. Therefore, the study is representative of the situation of children in Irish schools.

### Data collection

The EDI was used to measure child development at school entry age. It is a teacher completed questionnaire based on five months observation of the children from the date when they start school, and was, therefore, implemented in the latter half of the first year of formal education. Prior to completing the questionnaires, the teachers were given a short training and each issued with an EDI guide book. Children were not present when the questionnaire was completed and no individual identifiers were recorded. Passive consent was used in line with EDI studies conducted in Canada. The class teacher distributed an information letter to all parents two weeks before the study commenced. This contained detailed information on the study and parents were asked to contact the school if they did not want their child included (in total seven parents opted for their child not to be included). A form ID was assigned to each child which was used on both the EDI and Parental Questionnaire [16].

### Ethics statement

Ethical approval for the study was granted by the Clinical Ethics Committee of the Cork Teaching Hospitals. Passive consent (i.e. parents were given information on the study and asked to contact the school if they did not want their child included) was used as children were not present when the questionnaire was completed and no individual identifiers were provided to the research team. This is in line with international best practice in EDI studies [19].

### Parental questionnaire

In 2003 the Offord Centre developed and tested a parental questionnaire to complement the results of the EDI and provide a deeper population level context to the lives of children [20]. We adjusted the questionnaire to suit the Irish context and incorporated questions from the Growing Up in Ireland study [21] and the SLAN Study of Lifestyle, Behaviour and Nutrition in Ireland [22].

The parental questionnaire provided contextual data on many aspects of the children’s lives which have been described elsewhere [16]. However, in this study we were specifically interested in and only used data collected on utilisation of developmental support services.

The parental questionnaires were administered at the same time as the EDI and were distributed in school bags or homework folders. Each parental pack contained a letter of explanation, questionnaire (again with no individual identifier) and a blank envelope in which to return the questionnaire sealed to the school. Parents were reassured that the envelope would not be opened at the school. Data from the parental questionnaires was linked to the teacher filled questionnaire using the Form ID number and crosschecked using the recorded date of birth and gender. Questions were constructed in a Likert type response format - yes, no or three to five response options.

### Independent variables

**Figure 1:**
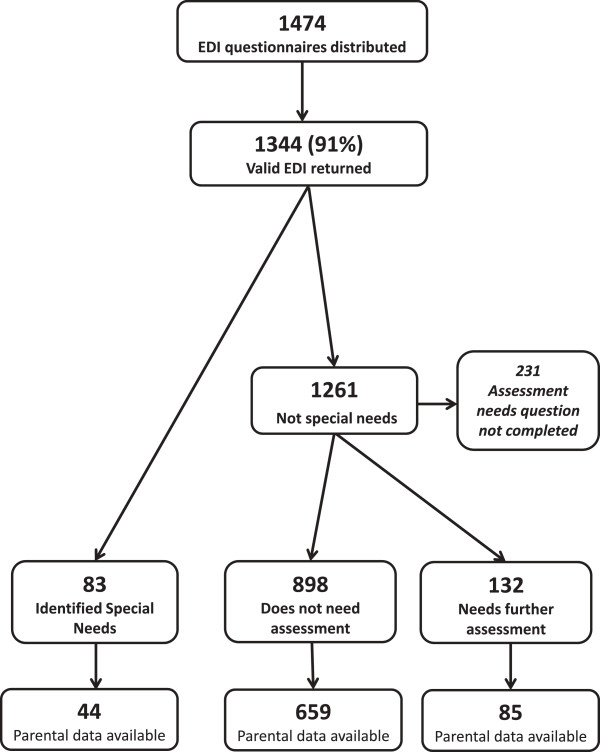
Participant flow chart.

For the purposes of this study three specific groups of children were identified and compared (see Figure 1). These were:

1 Children with special needs

Children in the ‘special needs’ group refers to those who had been identified as needing special assistance in the classroom through the nationally recognised assessment process. In Ireland this is defined as having a ‘Special Education Condition’ which has been recognised through a standardised assessment procedure [23]. In Section 1 of the EDI questionnaire teachers reported on whether the child had a special need identified through the above process. This did not seek the teacher’s opinion only information on whether the child had already received this designation.

2 Needs further assessment

Children who needed further assessment were those who had not been identified as having a Special Educational Condition through the standardised national assessment process but whom the teacher, based on her observation in the classroom, believed were in need of assessment. As part of the EDI questionnaire the teacher was asked whether, in her opinion, the child needed assessment.

3 Typically developing children

This refers to children who did not have a previously identified special need and who were not deemed by the teacher as needing further assessment.

### Dependant variables

Children in the three groups outlined above were compared using a number of variables. Comparisons were primarily made on EDI mean scores and vulnerability rates but also in relation to type of impairment, services accessed and residence in an area of deprivation/affluence. Data on EDI scores and type of impairment were obtained from the EDI questionnaire. Data on services accessed came from the parental questionnaire and data on area-level deprivation from the Irish National Deprivation Index for Health and Health Service Research 2013 (SAHRU Index) [24].

The child’s age was calculated using their date of birth and the date on which the form was completed and reported in years and months. Children for whom English was a second language (ESL) were those reported by the teacher to have a first language other than English.

#### EDI scores

EDI scores were calculated for each developmental domain i.e. Physical Health and Well-being; Social Competence; Emotional Maturity; Language and Cognitive Development; and Communication Skills and General Knowledge. All questions had either a binary or 2 or 3 point Likert type response format (yes, no, don’t know; very true, sometimes or somewhat true, never or not true, don’t know). All responses had a score of 0 to 10 (2 point answers were scored 0 and 10; 3 point answers were scored 0, 5 and 10). 'Don’t know' responses were not scored. If 30% of questions in any domain were not scored, that domain is not included. If more than one domain was excluded then that child’s score was not considered valid and excluded from the study. Domain scores referred to the child’s mean score in that domain - ranging between 0 and 10. Higher scores indicated better results.

#### Vulnerability rate

Children who scored in the lowest 10% of the study population in one or more of the five domains of the EDI were classed as ‘vulnerable’ [25]. Each domain was scored separately as children who were vulnerable in one area could not compensate through competence in another. Individual vulnerability was not reported rather vulnerability rates, expressed as percentages are used. In the absence of an Irish normative sample, to ensure the validity of the cut-off points, data was also scored against Canadian normative data. There was a 99% correlation between ‘vulnerability’ using the Irish and Canadian cut-off points. In four of the five domains there was 100% correlation between vulnerability using the Irish and Canadian cut-off points. Moreover, the EDI is a well validated instrument on which extensive psychometric testing has been conducted in both in Canada and Australia [17,19,25-27]. In the current study the EDI had good internal consistency by domains with Cronbach's α of between 0.8 and 0.96.

#### Impairment (specific problems)

In addition to questions aimed at assessing child development a section of the EDI questionnaire focused on specific problems. The teacher was asked whether the child had any impairment which influenced their ability to do regular classroom work and also whether s/he felt that the child needed further assessment.

Impairment referred to seven categories of problems that influenced the child’s ability to do school work in a regular classroom. These were listed on the EDI questionnaire, namely: physical impairment, visual impairment, hearing impairment, speech impairment, learning disability, behaviour problem or emotional problem. These were based on difficulties experienced by the child, not diagnosis. If children experience difficulty in more than one category, each was included.

#### Services accessed (parental report)

This information was obtained from the parental questionnaire. Parents were asked if their child had received help from any of a list of seven development support services: speech and language services; blind or low vision services; occupational of physical therapy; hearing services; programmes/ services for behavioural issues; programmes/ services for developmental issues; or mental health programmes/services. Parents were only asked if the child had ever ‘received help’ from the service and information was not included regarding the nature or extent of the support received from that service.

#### Area-level deprivation

The Irish National Deprivation Index for Health and Health Service Research 2013 (SAHRU Index) was used as a measure of deprivation. The index is based on a score calculated at the level of Electoral Division (3409 EDs in Ireland) using principal components analysis from a weighted combination of four indicators from the 2011 census, namely unemployment, low social class, local authority housing and no car [24]. Children were identified as residing in one of five quintiles ranging from most to least deprived based on their electoral division.

### Data analysis

Data analysis was conducted using SPSS. Initial scoring of the EDI data was conducted at the Offord Centre for Child Studies as part of the licensing agreement but all further analysis was conducted in University College Cork. Children were categorised into three groups, as outlined above. The mean scores in each of the five domains of development measured using the EDI were compared across the three groups of children using analysis of variance (ANOVA). As equality of variance could not be assumed, we used Tamhane’s T2 post hoc test to evaluate the mean difference between the groups. Residuals were tested for normal distribution.

## Results

EDI questionnaires were distributed to teachers of 1474 children in their first year of formal education in 47 schools (see Figure 1). A total of 1344 (91%) were completed and valid, 52.3% of which related to boys. Of the 1344 children, 83 (6.2%) had previously been identified as having special needs, the majority of whom (68%) were boys. A further 132 children (10%) were judged by the teacher to need further assessment. Again, boys predominated at 66%. There was no significant difference in the mean age between typically developing children, children who had an identified special need and the third group of children who were classed by the teacher as in need of further assessment. Demographic characteristics of the study population are outlined in Table 1.

**Table 1:** Demographic characteristics and mean scores on each EDI domain by special needs or needs further assessment

	**Typically developing**	**Special needs**	**Needs further assessment**
Number (% total population)*	898 (67)	83 (6)	132 (10)
% Boys	53	68	66
Age in years Mean (SD)	5.39 (.40)	5.55 (.52)	5.37 (.43)
% English as a second language	11	17	15
Vulnerable in one or more domain	17%	78%	69%
**Domain scores**	**Mean (SD)**	**Mean (SD)**	**Mean (SD)**
Physical well-being	8.99 (1.21)	6.48 (2.24)	7.13 (1.92)
Social competence	8.47 (1.66)	5.91 (2.18)	6.37 (2.01)
Emotional maturity	7.98 (1.44)	5.94 (1.82)	6.17 (1.81)
Language and cognitive development	8.96 (1.50)	6.54 (2.68)	7.16 (2.37)
Communication and general knowledge	7.91 (2.53)	3.82 (2.98)	4.54 (2.83)

*Data on assessment needs were not available on 231 children who did not have a Special Need.

### Developmental vulnerability

The study showed that 27% of children in the study population were developmentally vulnerable (i.e. in the lowest 10% of the population in at least one domain) at school entry age. The vulnerability rate rose to 78% among children with an identified special need and 69% among children who did not have a special need but whom the teachers identified as needing further assessment. There was a strong correlation between vulnerability on the EDI and needing further assessment (correlation coefficient = 0.379, p < 0.001).

### Mean scores for each group

**Figure 2:**
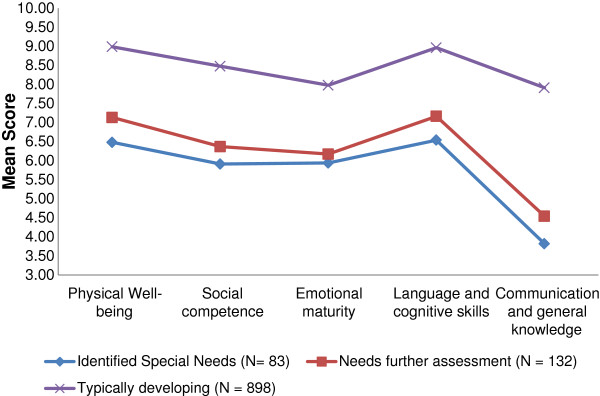
Mean domain scores by special needs status.

Typically developing children had high mean scores across all domains (Table 1) and were, therefore, more likely to be developmentally ready to engage in school than those children who were identified with special educational needs or in need of further assessment. Mean scores across all five domains of development for each of the three groups are outlined graphically in Figure 2.

When the mean scores in each domain were compared across the three groups using ANOVA there was a significant difference between the score of the typically developing group and each of the other two groups. However, there was no significant difference between the children with identified special needs and those needing further assessment (see Table 2). As test showed that equality of variance could not be assumed, Tamhane was used to examine the mean difference. Residuals were tested and shown to be normally distributed.

**Table 2:** Difference in mean scores between groups

**Domain**	Groups compared*	**Mean difference**	**Sig.**	**95% C.I.**
Physical well-being	Typically developing vs needs further assessment	-1.86	.000	(-2.28 to -1.43)
	Typically developing vs Special needs	-2.51	.000	(-3.13 to -1.88)
	Needs further assessment vs Special needs	-.65	.101	(-1.39 to .09)
Social competence	Typically developing vs needs further assessment	-2.11	.000	(-2.55 to -1.67)
	Typically developing vs Special needs	-2.57	.000	(-3.16 to -1.97)
	Needs further assessment vs Special needs	-.46	.331	(-1.17 to .26)
Emotional maturity	Typically developing vs needs further assessment	-1.81	.000	(-2.2 to -1.41)
	Typically developing vs Special needs	-2.04	.000	(-2.54 to -1.54)
	Needs further assessment vs Special needs	-.23	.735	(- .85 to .38)
Language and cognitive development	Typically developing vs needs further assessment	-1.80	.000	(-2.31 to -1.29)
	Typically developing vs Special needs	-2.42	.000	(-3.15 to -1.70)
	Needs further assessment vs Special needs	-.63	.228	(-1.49 to .24)
Communication skills and general knowledge	Typically developing vs needs further assessment	-3.37	.000	(-4.0 to -2.74)
	Typically developing vs Special needs	-4.09	.000	(-4.92 to -3.27)
	Needs further assessment vs Special needs	-.72	.218	(-1.71 to .26)

*One-way ANOVA.

### Impairment (specific problems)

One quarter (25%) of all children with identified special needs had a physical impairment. Almost half (45%) had a speech impairment, 39% a learning disability, 28% emotional and 24% behavioural problems. Relative to children with identified special needs, those designated as needing further assessment were less likely to have physical disability (5%). However, 39% were deemed by the teacher to have difficulties with speech and language, 22% learning difficulties, 19% emotional problems and 21% behavioural problems (Table 3).

**Table 3:** Type of Impairment* among children with Special Needs or Needing further assessment

	**Physical disability**	**Visual impairment**	**Hearing impairment**	**Speech impairment**	**Learning disability**	**Emotional problem**	**Behavioural problem**
	**%**	**%**	**%**	**%**	**%**	**%**	**%**
Identified special needs	25.3	6.0	6.0	44.6	38.6	27.7	24.1
Needs further assessment	5.3	3.0	1.5	39.4	22.0	18.9	21.2

*Teachers were asked to identify if the children had an impairment which prevented them from fully participating in classroom activities.

### Social gradient

**Figure 3:**
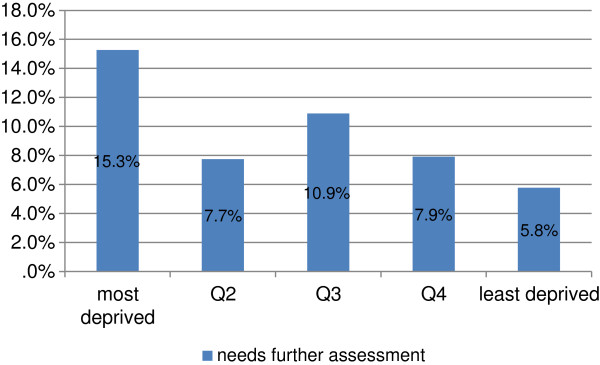
Percentage of children requiring further assessment by deprivation quintile.

There was evidence of a social gradient among children needing assessment (Figure 3). Over 15% of children living in the most deprived area quintile were deemed by the teacher as needing further assessment compared to 5.8% of those living in the most affluent quintile.

### Services accessed

Information on services with which the children had contact was available on a subset of 963 children on whom parental questionnaires were returned. Of this subset, 44 (4.6%) were identified as special needs and 85 (8.8%) were deemed to need further assessment. Children for whom parental questionnaires were returned also had significantly higher mean scores in all developmental domains and were less likely to be scored as vulnerable on the EDI than those for whom parental data were not available [16].

The majority of children who had special needs (85%) had accessed at least one support service. However, this was not the case for children who were identified as needing further assessment of whom less than half (48%) had accessed services. The services most commonly accessed by this group were Speech and Language services (36.6%) and Hearing Services (19%). They had very limited access to services for behavioural issues (5.1%), developmental issues (5.2%) or mental health (0). Services accessed are outlined fully in Table 4.

**Table 4:** Services accessed (based on parental reports)

	Special needs (N = 44)*	Needs further assessment (N = 85)*
	**%**	**%**
Speech and language services	65.9	36.6
Blind or low vision services	9.8	2.5
Occupational or physical therapy	61.0	5.1
Hearing services	29.3	19.0
Services for behavioural issues	27.5	5.1
Services for developmental issues	37.5	5.2
Mental health programmes	5.1	0

*Parental data was available only on a sub-set of 963 children.

## Discussion

This paper illustrates that children who have special educational needs are at a greater risk of not being ready to engage in formal education. However, the majority (80%) do have access to support services. Of concern are the 10% of children in the study who were deemed by their teacher to be in need of further assessment. These children showed an equivalent level of vulnerability across all domains of development to the children with special needs but less than half had accessed any services. Learning difficulties, behavioural and emotional problems were prominent among this group. Yet they were more likely to have accessed hearing services than those which deal with their identified problems.

Children with a physical impairment were more likely to have had their special need identified. Only 5% of those who needed further assessment had a physical disability. Similar results from an evaluation of special needs referral in a large Head Start programme showed that children with emotional or behavioural problems were less likely to be referred for assessment [28]. Failure to support children experiencing difficulties in the early years can lead to low self esteem and a sense of worthlessness that can have a profound effect on the mental, social, emotional and cognitive development for the child concerned.

A recent report by the by the National Council for Special Education (NCSE) in Ireland highlighted a number of issues regarding the assessment of special educational needs in Ireland [13]. The assessment process is a continuum from the identification of class room based supports or in-school supports as assessed by teachers (for children with mild challenges) to external assessment of additional support needs where a child is experiencing more profound difficulty. The report raised concerns regarding the link between resource allocation and the diagnosis of a particular category of disability. It appears as imperative that a child has a label prior to any entitlement to additional supports. Some conditions are easier to detect than others, for example severe autism, Down’s syndrome, cerebral palsy and other visible conditions. It is the so called ‘hidden disabilities’ that also need early detection if the child is to be afforded every chance at a productive life. Indeed the necessity of a definitively diagnosed disability prior to recognition of special needs status is questionable [29].

In the context of truly inclusive education, a strong focus on participation, functioning and the educational environment as opposed to diagnosis of particular conditions would ensure that the needs of all children are met [30,31]. The NCSE report states that while school principals have responsibility for seeking assessments when they consider it necessary, very often the number of assessments available to schools is limited resulting in long waiting lists and subsequent delays in allocating the required resources to support the child’s learning needs. Parents can seek private assessments but these are expensive and therefore not assessable to children in families with limited financial resources. Where parents can afford to pay for private assessment, the child will benefit from more timely allocation of resources and support [15]. The social gradient in the number of children identified as requiring assessment in this study supports the assertion.

The strong link between assessment, identification of a particular ‘condition’ and allocation of resources may not serve the best interests of the child. The assessment should involve the development of an individual educational plan that builds on the child’s strengths and supports their needs [10]. However, in the pressure to provide a diagnosis with resultant resources, the need for a process which is inclusive of the views of teachers and parents with the objective of developing an individually appropriate plan may be overlooked. This study shows that teachers are well placed to correctly identify those children requiring additional support at a very early age.

The study demonstrates that teacher observation is an effective means of identifying children who have a level of impairment which prevents them from fully participating in their educational environment. This is supported by evidence from studies of teacher-completed rating scales [32]. Moreover, a recent qualitative study conducted in Ireland found that teachers felt that they could play a more active role in the assessment process [15]. A multi-disciplinary approach towards children with special educational needs could integrate teacher observation with other approaches to assessment and support a model of education which would be inclusive of the needs of every child.

### Limitations

This study of early development outcomes was conducted with 1344 children in 47 schools and has examined special educational needs in the context of a typically developing population. However, as only 132 children needed further assessment and only 83 were identified as having special educational needs, it was not possible to examine in depth the underlying factors which may determine why some children’s support needs are not identified or met. Factors at the individual and family level that may contribute to developmental vulnerability are not explored in this paper but have been previously published [16].

Parents were asked to recall which of the services their children had attended from a list provided. This may have led to some degree of recall bias. Moreover, parents were not asked if the child received the necessary support from these services therefore we do not know to what extent the needs of the children were addressed by accessing these services.

## Conclusions

A small but significant number of children have not had their needs adequately assessed. Teacher observation is an effective means of identifying children with a level of impairment which prevents them from fully participating in their educational environment and could be integrated into a multi-disciplinary approach to meeting the needs of all children.

## Competing interests

The authors do not have any competing interests financial or otherwise with regard to this manuscript.

## Authors’ contribution

MC conducted the data collection and analysis and drafted the manuscript. DB conducted the data collection and assisted with drafting the manuscript. AS contributed to overall project and study design, data analysis and edited and approved the manuscript. IJP was responsible for the overall conceptualisation of the project, study design and edited and approved the manuscript. All authors read and approved the final manuscript.

## Pre-publication history

The pre-publication history for this paper can be accessed here:

http://www.biomedcentral.com/1471-2431/14/52/prepub
